# Interactive effects of asparagine and aspartate homeostasis with sex and age for the risk of type 2 diabetes risk

**DOI:** 10.1186/s13293-020-00328-1

**Published:** 2020-10-22

**Authors:** Hui-Huan Luo, Xiao-Fei Feng, Xi-Lin Yang, Rui-Qin Hou, Zhong-Ze Fang

**Affiliations:** 1 Department of Toxicology and Sanitary Chemistry, School of Public Health, Tianjin Medical University, Tianjin, 300070 China; 2 Department of Epidemiology and Biostatistics, School of Public Health, Tianjin Medical University, Tianjin, China; 3 Tianjin Key Laboratory of Environment, Nutrition and Public Health, Tianjin, China; 4 Department of Blood Transfusion, Peking University People’s Hospital, Beijing, China

**Keywords:** Asparagine and aspartate homeostasis, Sex, Age, Type 2 diabetes risk interaction

## Abstract

**Background:**

Asparagine and aspartate homeostasis are linked with type 2 diabetes (T2D). This study aimed to explore whether asparagine and aspartate metabolism interacted with sex and age to increase the risk of T2D.

**Methods:**

From 27 May 2015 to 3 August 2016, we consecutively retrieved 1032 T2D patients and 1522 subjects without T2D from a tertiary care hospital in Liaoning, China. Restricted cubic spline nested in the logistic regression was used to draw odds ratio curves of plasma asparagine to aspartate ratio for T2D by sex and age. Cut-off point was selected where curves went apart, indicating possible interaction. Addictive interactions of asparagine to aspartate ratio with sex or age and secondary interaction with copresence of unfavorable sex and age were further estimated using relative excess risk due to interaction (RERI), attributable proportion due to interaction (AP), and synergy index (S).

**Results:**

Ratio of asparagine to aspartate > 1.5 was associated with elevated risk of T2D (OR 7.99, 95%CI 5.50 to 11.6), which was enhanced by female gender to 13.6, (95%CI 8.10–22.9) and by > 50 years of age to 28.7 (14.6–56.3), with significant additive interactions. There was a significant secondary-interaction of copresence of female sex and > 50 years of age with high asparagine to aspartate ratio for increased T2D risk with the OR being further increased to 34.4 (20.5–57.5).

**Conclusions:**

High asparagine to aspartate ratio was associated with markedly increased risk of T2D, which was further amplified by either female gender or > 50 years of age, and especially both.

**Supplementary information:**

**Supplementary information** accompanies this paper at 10.1186/s13293-020-00328-1.

## Background

There is a small but significant sex differences in the prevalence of type 2 diabetes (T2D) globally [1–3]. Cross-sectional and prospective investigations all showed that males are more likely to have insulin resistance and to have higher prevalence of diabetes in late adolescence up to midlife than females [4, 5]. Females experienced a sharply increased risk of diabetes after the menopause, resulting in nearly equal prevalence of T2D as males in older life [1, 2, 6]. The related pathophysiology includes but is not limited to gene, diet, physical activity, and sex hormone fluctuance [7–9]. Despite of extensive research on this issue, the underlying mechanisms remain elusive [1]. Nevertheless, unraveling sex-specific mechanisms on T2D onset, especially in different life span, is of importance for personalized diabetes management. With advances in metabolomics research and techniques, metabolite profiling has the potential to identify pathways associated with metabolic risk and to provide novel insight into this scientific issue.

Asparagine and aspartate can be converted to each other by corresponding enzymes with metabolism fluctuation. Asparagine synthetase expresses in many tissues especially pancreas and catalyzes the conversion of aspartate to asparagine in an ATP-dependent way [10, 11]; asparaginase can catalyze the conversion of asparagine to aspartate [12]. As a critical source of energy, building blocks of protein synthesis and signaling molecules, asparagine and aspartate were found to be linked to metabolic disorder traits and metabolic pathways in animal and human studies: knockdown of asparagine synthetase in rat liver-reduced plasma glucose [13]; adenosine monophosphate-activated protein kinase (AMPK) α phosphorylation and the mammalian target of rapamycin complex 1 (mTORC1), two key pathways participating in metabolism, were sensitive to asparagine concentration [14, 15]. In this connection, Framingham Heart Study showed that asparagine was inversely associated with fasting insulin while aspartate was inversely associated with fasting glucose [16]; overweight/obese pregnant women had a higher total amount of asparagine and aspartate [17]. Interestingly, these traits and pathways were previously found to be associated with crucial mechanisms which contributed to sex difference in different age groups, for example, higher insulin resistance in males than females before older life [5] and sudden deficiency of estrogens due to menopause at ~ 50 years of age in females [18–20]. Hence, it was possible that asparagine and aspartate homeostasis might help interpret these sex-specific mechanisms.

Based on the above observations, we hypothesize that asparagine and aspartate homeostasis is linked to the sex difference in the prevalence of diabetes. Therefore, this hospital-based study aimed to examine additive interactions of high ratio of asparagine and aspartate with female gender and > 50 years of age on the risk of T2D.

## Methods

### Research design and study patients

The study patients and methods were described previously [21]. From 27 May 2015 to 3 August 2016, a total of 71,020 patients were hospitalized in Liaoning Medical University First Affiliated Hospital (LMUFAH). Among them, 1032 consecutive patients were diagnosed with T2D and had complete information on height, weight, and blood pressure. Non-T2D subjects were recruited from hospital’s physical examination center where individuals had regular health examination. One thousand five hundred twenty-two of them had metabolomic profiles measured and were included in this analysis. All subjects were over 18 years old. T2D was diagnosed according to the 1999 World Health Organization’s criteria or use of anti-diabetic drugs [22]. The Ethics Committee for Clinical Research of LMUFAH approved the ethics of the study, and informed consent was waivered by the above ethics committee due to the retrospective nature of the study, which is consistent with the Declaration of Helsinki.

### Data collection and definitions

Clinical information including demography, anthropometry, laboratory parameters, medications, and disease status was extracted from electronic medical system (EMS) retrospectively. Age was calculated automatically by EMS as difference between admission year and birth year. Body mass index (BMI) was calculated as weight in kilograms divided by squared height in meters. According to the recommended Chinese criteria [23], BMI was categorized into four classes: underweight (< 18.5 kg/m^2^), normal weight (18.5 to 24 kg/m^2^), overweight (> 24 kg/m^2^), and obesity (≥ 28 kg/m^2^). Systolic blood pressure (SBP) over 140 mmHg, high-density lipoprotein cholesterol (HDL-C) less than 1 mmol/L in men and 1.3 mmol/L in women, triglyceride over 1.7 mmol/L, low-density lipoprotein cholesterol (LDL-C) over 2.6 mmol/L were defined as abnormal [24]. Use of oral anti-diabetic drugs and insulin, angiotensin-converting enzyme inhibitors (ACEIs), angiotensin receptor blockers (ARBs), and other anti-hypertensive drugs, statins, and other lipid-lowering drugs in hospital was documented. Complications were extracted from the electronical database, including coronary artery disease, stroke, diabetic retinopathy, and diabetic nephropathy.

### Measurements of serum asparagine and aspartate

Details of the amino acid assessment method were published previously [25]. Eight-hour fasting blood was taken and stored as dried blood spot. Eight-hour fasting blood was mostly collected in the morning. As no reliable evidence has suggested circadian rhythm in conversion of asparagine to aspartate, time of sampling was unlikely to affect the levels of amino acids. Amino acids quality control (QC) standards used in our study were provided by Chromsystems (Grafelfing, Germany). LC-MS/MS analysis was performed with AB Sciex 4000 QTrap system (AB Sciex, Framingham, MA, USA). Analyst v1.6.0 software (AB Sciex) was used for system control and data collection. ChemoView 2.0.2 (AB Sciex) was used for data preprocessing.

### Statistical analysis

We first compared difference of clinical and biochemical characteristics of participants between T2D and non-T2D. For continuous variables, Q-Q plot was used to checked normality. Data with normal distribution was expressed as means (standard deviations) while data with skewed distribution was expressed as medians (interquartile ranges). Non-paired Student’s *t* test (or Mann-Whitney *U* test when appropriate) was used to measure the difference; for categorical variables, data was presented as frequencies (percentage) and chi-square test (or fisher test if appropriate) was used for difference comparison.

Age was stratified into a binary variable at 50 years, and > 50 years of age in female roughly represented the postmenopausal period. Restricted cubic spline (RCS) nested in the logistic regression was also performed to examine the full-range associations between age with T2D risk and to ascertain the selected cut-off point at 50 years of age [26]. The associations between asparagine or aspartate alone with T2D were examined with RCS too. Next, to visualize potential interaction between asparagine to aspartate ratio with sex or age, we also performed RCS to examine the full-range associations between asparagine to aspartate ratio with T2D in different sex and age groups which derived from cut-off points chosen above. That OR curves turned apart and became unparallel may imply an additive interaction. The point of asparagine to aspartate ratio where curves turned apart and unparallel was selected as the cut-off point to categorize the ratio for further analysis.

Then, we formally tested the additive interaction by calculating relative excess risk due to interaction (RERI), attributable proportion due to interaction (AP), and synergy index (S) [27]. RERI > 0, AP > 0, or S > 1 indicates biological interaction. First, we used univariable and multivariable logistic regression models to obtain odds ratio (OR) and 95% confidence (CI) of asparagine, aspartate, asparagine to aspartate ratio, sex, and age (all as categorical variables) for T2D. Asparagine to aspartate ratio (> 1.5 and ≤ 1.5 μmol/L, sex, age (< 50 and ≥ 50 years, BMI (< 18.5, 18.5 ~ 24.0, 24.0 ~ 28.0 and ≥ 28.0 kg/m^2^), SBP (< 140 and ≥ 140 mmHg), LDL-C (< 2.60 and ≥ 2.60 mmol/L), HDL-C (< 1.00 mmol/L in male or < 1.30 mmol/L in female as low level and ≥ 1.00 in male or ≥ 1.30 in female as high level) and triglyceride (< 1.70 mmol/L and ≥ 1.70 mmol/L) were included in multivariable analysis. Through steps above, high asparagine, low aspartate, high asparagine to aspartate ratio, female and age over 50 years old were identified as risk factors of T2D (see Table 2); second, to estimate the biological interaction between asparagine to aspartate ratio and age or sex, we created four variables (see Table 2): (1) asparagine to aspartate ratio ≤ 1.5 μmol/L and age < 50 years (or male) (as reference); (2) asparagine to aspartate ratio ≤ 1.5 μmol/L and age ≥ 50 years (or female); (3) asparagine to aspartate ratio > 1.5 μmol/L and age < 50 years (or male); (4) asparagine to aspartate ratio > 1.5 μmol/L and age ≥ 50 years (or female). Confounders listed above were also adjusted in multivariable addictive models.

We further tested the second-order interaction between high asparagine to aspartate ratio and coexistence of female sex and > 50 years of age. The four variables were created as combination of copresence of female sex and > 50 years of age and high asparagine to aspartate ratio: (1) female and > 50 years of age = no plus low asparagine to aspartate ratio (use as reference); (2) female and > 50 years of age = yes plus low asparagine to aspartate ratio; (3) female and > 50 years of age = no plus high asparagine to aspartate ratio; (4) female and > 50 years of age = yes plus high asparagine to aspartate ratio (see Table 3). Likewise, RERI, AP, and S were used to measure biological interactions.

To exclude potential influence of diabetes-related parameters on association between asparagine to aspartate ratio and T2D, especially in the context of gender, we included patients without diabetes complications or use of anti-diabetic medications in the analysis and repeated logistic regression and addictive interaction analysis; to avoid some possible bias from different age distribution for each gender or different gender distribution for each age groups, we also compared male and female gender in different age groups, as well as age over and below 50 years old in each gender.

Partial Pearson correlation (data with normal distribution ) or Spearman correlation (data with skewed distribution) analysis was used to test correlations between asparagine to aspartate ratio and available diabetes traits, i.e., BMI, SBP, triglyceride, LDL-C, HDL-C, HbA1c, and duration of diabetes while adjusted for age and sex.

All analysis was performed using SAS version 9.4 (SAS institute Inc., Cary, NC, USA) and R version 3.6.0. *P* values of < 0.05 were considered statistically significant.

## Results

### Characteristics of the study population

There were 1032 T2D and 1522 non-T2D subjects. The mean age of patients with T2D was 57.2 (SD 13.8) years and the mean age of subjects without was 46.4 (13.7) years. Of them, 53.2% of T2D and 74.3% of non-T2D were males. Patients with T2D had a mean HbA1c of 9.60% (SD 2.38%). Duration of diabetes was 5 (IQR 0–10) years. Patients with T2D were older and had higher SBP, triglyceride, tyrosine, asparagine, and asparagine to aspartate ratio than subjects without T2D. Levels of HDL-C, LDL-C, and aspartate were lower in T2D than in non-T2D. BMI was similar between T2D and non-T2D. The prevalence of diabetes complications and drug use were shown in Table 1.

**Table 1:** Clinical and biochemical characteristics of participants according to T2D

Characteristics	Non-T2D	T2D	*P* value
No. of subjects	1522	1032	
Duration of diabetes, years		5 (0–10)	
Age, years	46.4 ± 13.7	57.2 ± 13.8	< .0001
≥ 50 years old	640 (42.1)	785 (76.1)	< .0001
Male sex	1131 (74.3)	549 (53.2)	< .0001
BMI, kg/m^2^	25.4 ± 3.5	25.3 ± 3.9	0.3338
BMI < 18.5	23 (1.5)	27 (2.6)	0.2012
BMI ≥ 18.5 and < 24	504 (33.1)	354 (34.3)	
BMI ≥ 24 and < 28	653 (42.9)	430 (41.7)	
BMI ≥ 28	342(22.5)	221 (21.4)	
SBP, mmHg	130.9 ± 17.2	140.4 ± 24.0	< .0001
HDL-C, mmol/L	1.55 ± 0.35	1.08 ± 0.35	< .0001
< 1.00 in male or < 1.30 in female	123 (8.1)	785 (76.1)	< .0001
LDL-C, mmol/L	3.06 ± 0.70	2.89 ± 1.01	< .0001
LDL-C > 2.60 mmol/L	1127 (74.1)	434 (42.1)	< .0001
Triglyceride, mmol/L	1.51 (1.02–2.35)	1.67 (1.11–2.38)	0.0126
Triglyceride > 1.70	644 (42.8)	361 (48.5)	0.0097
HbA1c, %		9.60 (2.38)	
Asparagine, μmol/L	70.68 (59.77–84.12)	74.85 (61.98–89.50)	< .0001
> 88, μmol/L	303 (19.9)	280 (27.1)	< .0001
Aspartate, μmol/L	42.54 (29.67–58.01)	28.37 (20.91–37.56)	< .0001
< 65, μmol/L	1275 (83.8)	1014 (98.3)	< .0001
Asparagine: aspartate	1.72 (1.26–2.37)	2.60 (1.99–3.55)	< .0001
≤ 1.5 μmol/L	578 (38.0)	87 (8.4)	< .0001
> 1.5 μmol/L	944 (62.0)	945 (91.6)	
Macrovascular complications			
Prior CAD		210 (20.4)	
Prior stroke		199 (19.3)	
Microvascular complications			
Diabetic retinopathy		162 (15.7)	
Diabetic nephropathy		187 (18.1)	
Diabetes medications			
Oral anti-diabetic drugs		564 (55.1)	
Insulin		770 (74.8)	
Statins		369 (35.9)	
Other lipid-lowering drugs		23 (2.2)	
ACEIs		135 (13.1)	
ARBs		134 (13.0)	
Other anti-hypertensive drugs		309 (29.9)	

*Abbreviations*: *T2D* type 2 diabetes, *BMI* body mass index, *SBP* systolic blood pressure, *HDL-C* high-density lipoprotein cholesterol, *LDL-C* low-density lipoprotein cholesterol, *HbA1c* glycated hemoglobin, *CAD* coronary artery disease, *ACEI* angiotensin-converting enzyme inhibitors, *ARB* angiotensin receptor blockers

Data are means ± standard deviation (SD) or median (interquartile range [IQR]) or *n* (%)

*P* values were derived from independent-samples Student’s *t* test for normally distributed variables, Mann-Whitney *U* test for skewed distributions, chi-square test (or fisher test if appropriate) for categorical variables

### Addictive interaction of asparagine to aspartate ratio with age and sex for T2D

**Fig. 1:**
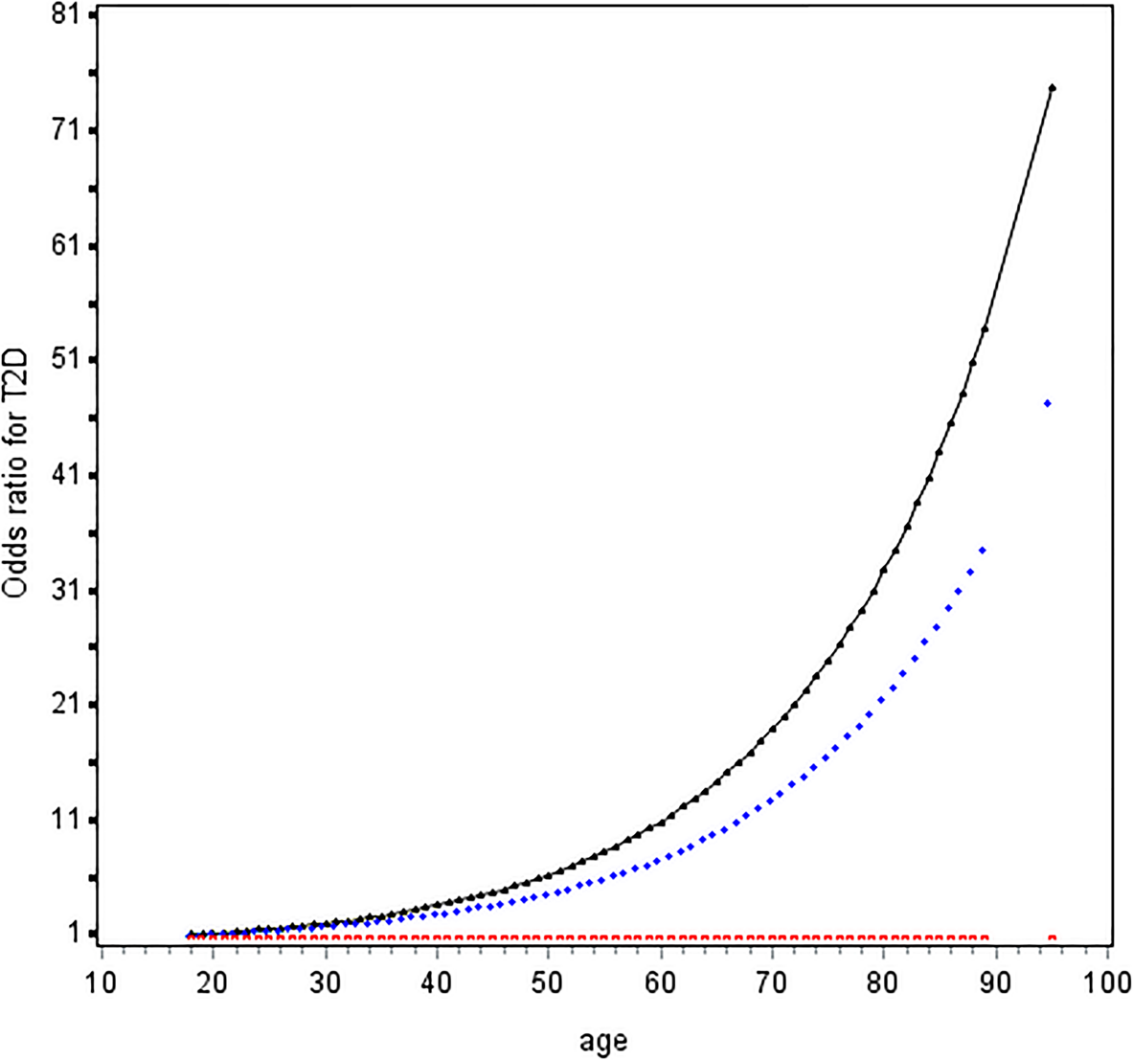
Odds ratio curves of age for T2D risk. Abbreviations: T2D, type 2 diabetes. The black curve was derived from univariable analysis, and the blue curve derived from multivariate analysis that adjusted for body mass index (< 18.5, 18.5 ~ 24.0, 24.0 ~ 28.0, and ≥ 28.0 kg/m2), systolic blood pressure (< 140 and ≥ 140 mmHg), low-density lipoprotein cholesterol (< 2.60 and ≥ 2.60 mmol/L), high-density lipoprotein cholesterol (< 1.00 mmol/L in male or < 1.30 mmol/L in female as low level and ≥ 1.00 in male or ≥ 1.30 in female as high level), and triglyceride (< 1.70 mmol/L and ≥ 1.70 mmol/L); The red curve stands for the reference level (i.e., the odds ratio for type 2 diabetes mellitus was 1)

**Fig. 2:**
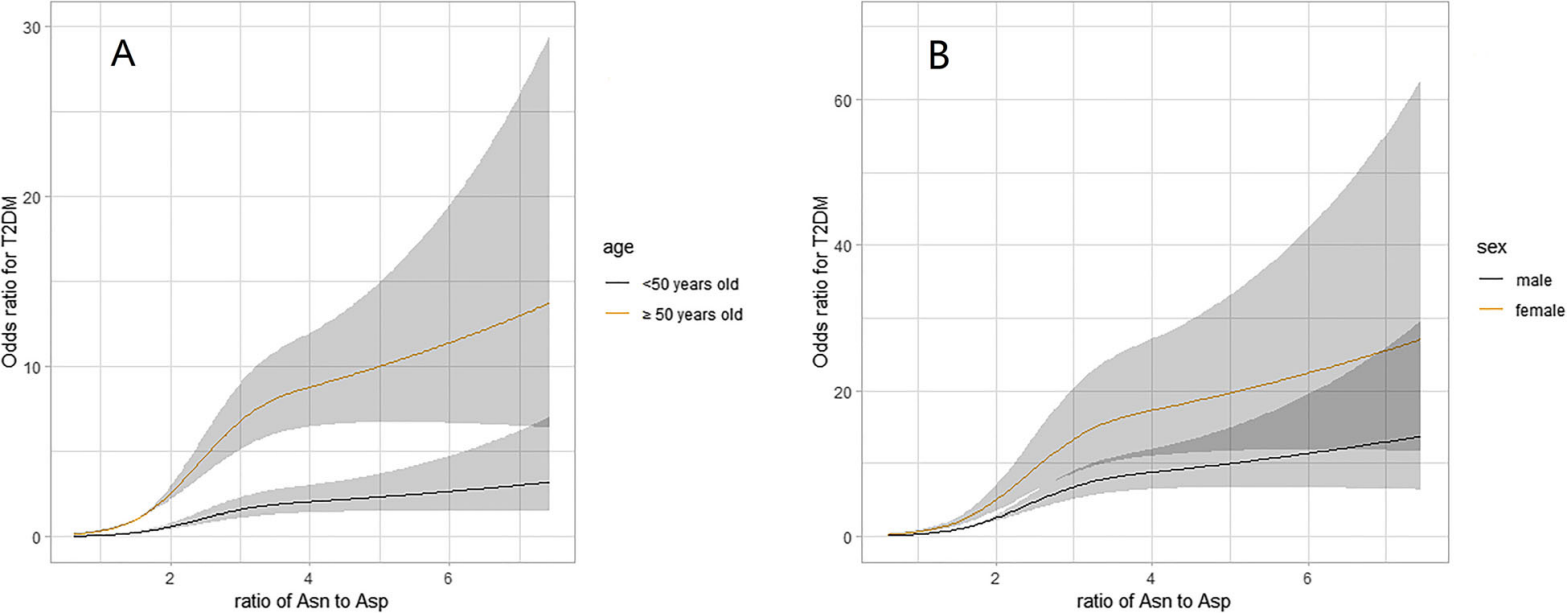
ORs curves of asparagine to aspartate ratio for T2D in different sex and age groups. Abbreviations: ORs, odds ratios; T2D, type 2 diabetes; Asn, asparagine; Asp, aspartate; Curves derived from multivariable analysis that adjusted for body mass index (< 18.5, 18.5 ~ 24.0, 24.0 ~ 28.0, and ≥ 28.0 kg/m^2^), systolic blood pressure (< 140 and ≥ 140 mmHg), low-density lipoprotein cholesterol (< 2.60 and ≥ 2.60 mmol/L), high-density lipoprotein cholesterol (< 1.00 mmol/L in male or < 1.30 mmol/L in female as low level and ≥ 1.00 in mal or ≥ 1.30 in female as high level), triglyceride (< 1.70 mmol/L and ≥ 1.70 mmol/L), and sex in **a** and age (< 50 and ≥ 50 years old) in **b**

Age was associated with T2D in a J-shape relationship (Fig. 1). The risk of T2D rapidly increased from 50 years upwards. OR (95%CI) of age ≥ 50 vs. < 50 years was 3.96 (2.99–5.24) in multivariable analysis (Table 2); asparagine was positively while aspartate was inversely associated with increased T2D risk non-linearly (Figure S1). Exposure to high asparagine or low aspartate led to 2.38-fold (95% CI, 1.77–3.21) and 6.33-fold (4.54–8.82) risk of diabetes compared to their counterparts, respectively (Table 2); Asparagine to aspartate ratio was associated with increased risk of T2D and at 1.5 upwards, the two curves in patients aged ≥ 50 years and < 50 years went apart (Fig. 2a) and became unparallel in both males and females (Fig. 2b). OR (95%CI) of high vs. low asparagine to aspartate ratio for T2D was 7.00 (5.27–9.30) in univariable analysis and 7.99 (5.50–11.6) in multivariable analysis. Female had 1.82-fold risk of male counterparts (Table 2).

**Table 2:** Interactive effects of asparagine and aspartate homeostasis with sex and age for T2D risk

	Univariable model			Multivariable model	
	OR (95% CI)	*P* value		OR (95% CI)	*P* value
Asn > 88 vs. ≤ 88, μmol/L	1.69 (1.38–2.08)	< 0.0001		2.38 (1.77–3.21)	< 0.0001
Asp < 65 vs. ≥ 65, μmol/L	11.7 (6.49–21.0)	< 0.0001		6.33 (4.54–8.82)	< 0.0001
Asn: Asp > 1.5 vs. ≤ 1.5	7.00 (5.27–9.30)	< 0.0001		7.99 (5.50–11.6)	< 0.0001
Age ≥ 50 vs. < 50 years old	4.62 (3.79–5.64)	< 0.0001		3.96 (2.99–5.24)	< 0.0001
Female vs. male	2.55 (2.12–3.06)	< 0.0001		1.82 (1.38–2.42)	< 0.0001
**Additive interaction model of asparagine to aspartate ratio and age** ^**a**^					
Asn: Asp ≤ 1.5 and age < 50 years old	Reference			Reference	
Asn: Asp ≤ 1.5 and age ≥ 50 years old	4.99 (2.60–9.60)	< 0.0001		3.43 (1.61–7.29)	0.0014
Asn: Asp > 1.5 and age < 50 years old	7.60 (4.16–13.9)	< 0.0001		7.10 (3.60–14.0)	< 0.0001
Asn: Asp > 1.5 and age ≥ 50 years old	37.0 (20.5–66.9)	< 0.0001		28.7 (14.6–56.3)	< 0.0001
Interaction measure	Estimates			Estimates	
RERI	25.4 (10.4–40.5)			19.2 (6.02–32.3)	
AP	0.69 (0.62–0.76)			0.67 (0.57–0.77)	
S	3.40 (2.64–4.39)			3.25 (2.32–4.55)	
**Additive interaction model of asparagine to aspartate ratio and sex** ^**b**^					
Asn: Asp ≤ 1.5 and male	Reference			Reference	
Asn: Asp ≤ 1.5 and female	2.09 (1.22–3.56)	0.0070		1.49 (0.76–2.93)	0.2676
Asn: Asp > 1.5 and male	6.46 (4.34–9.62)	< .0001		7.19 (4.41–11.7)	< .0001
Asn: Asp > 1.5 and female	21.2 (14.0–32.2)	< .0001		13.6 (8.10–22.9)	< .0001
Interaction measure	Estimates			Estimates	
RERI	13.7 (7.48–19.9)			5.95 (1.60–10.3)	
AP	0.64 (0.56–0.73)			0.44 (0.26–0.62)	
S	3.09 (2.35–4.05)			1.89 (1.32–2.71)	

*Abbreviations*: *T2D* type 2 diabetes, *Asn* asparagine, *Asp* aspartate, *OR* odds ratio, *CI* confidence interval, *RERI* risk due to interaction, *AP* attributable proportion due to interaction, *S* synergy index

Multivariable analysis adjusted for body mass index (< 18.5, 18.5 ~ 24.0, 24.0 ~ 28.0 and ≥ 28.0 kg/m^2^), systolic blood pressure (< 140 and ≥ 140 mmHg), low-density lipoprotein cholesterol (< 2.60 and ≥ 2.60 mmol/L), high-density lipoprotein cholesterol (< 1.00 mmol/L in male or < 1.30 mmol/L in female as low level and ≥ 1.00 in male or ≥ 1.30 in female as high level), triglyceride (< 1.70 mmol/L and ≥ 1.70 mmol/L), and sex in a or age (< 50 and ≥ 50 years old) in b

Significant elative excess risk due to interaction (RERI) > 0, attributable proportion due to interaction (AP) > 0 or synergy index (S) > 1 indicates a significant additive interaction

Using low asparagine to aspartate ratio and < 50 years of age as the reference, the OR was 3.43 (95% CI 1.61–7.29) for individuals who were ≥ 50 years of age and had a low asparagine to aspartate ratio; and 7.10 (95% CI 3.60–14.0) for individuals who were < 50 years of age and had a high asparagine to aspartate ratio, after adjusting for confounders. Coexistence of older age and high asparagine to aspartate ratio sharply increased the effect size to 28.7 (95% CI 14.6–56.3), with significant additive interaction (RERI 19.2, 95% CI 6.02–32.3; AR 0.67, 0.57–0.77; S 3.25, 2.32–4.55) (Table 2).

Similarly, using low asparagine to aspartate ratio and male as the reference and in multivariable analysis, the OR was 1.49 (95% CI 0.76–2.93) in patients who were female and had a low asparagine to aspartate ratio; and 7.19 (95% CI 4.41–11.7) in patients who were male and had a high asparagine to aspartate ratio. Being female and having a high asparagine to aspartate ratio had the highest OR, i.e., 13.6 (95% CI 8.10–22.9). The additive interaction was significant (RERI 5.95, 95% CI 1.60–10.3; AR 0.44, 0.26–0.62; S 1.89, 1.32–2.71) (Table 2).

### Second-order interaction of high asparagine to aspartate ratio and coexistence of female sex and older than 50 years old

There was a significant second-order additive interaction between high asparagine to aspartate ratio and coexistence of female gender and ≥ 50 years of age for risk of T2D (Table 3). Coexistence of female gender and ≥ 50 years of age greatly amplified the OR for T2D from 7.99 (5.50–11.6) to 34.4 (20.5–57.5). The additive interaction was highly significant (RERI 26.1, 95% CI 11.0–41.2; AP 0.76, 0.66–0.86; and S 4.58, 95% CI 2.96–7.08) in multivariable analysis.

**Table 3:** Secondary interaction of age and sex with asparagine and aspartate homeostasis for T2D

	OR (95% CI)	*P* value
**Univariable additive interaction model**		
Female and > 50 years of age = no plus Asn: Asp ≤ 1.5	reference	
Female and > 50 years of age = yes plus Asn: Asp ≤ 1.5	4.53 (2.57–7.98)	< .0001
Female and > 50 years of age = no plus Asn: Asp > 1.5	6.76 (4.70–9.71)	< .0001
Female and > 50 years of age = yes plus Asn: Asp > 1.5	52.9 (34.4–81.2)	< .0001
Interaction measure	Estimates	
RERI	42.6 (22.7–62.5)	
AP	0.81 (0.74–0.87)	
S	5.58 (3.90–8.00)	
**Multivariable additive interaction model**		
Female and > 50 years of age = no plus Asn: Asp ≤ 1.5	reference	
Female and > 50 years of age = yes plus Asn: Asp ≤ 1.5	2.78 (1.35–5.71)	0.0053
Female and > 50 years of age = no plus Asn: Asp > 1.5	6.50 (4.24–9.95)	< .0001
Female and > 50 years of age = yes plus Asn: Asp > 1.5	34.4 (20.5–57.5)	< .0001
Interaction measure	Estimates	
RERI	26.1 (11.0–41.2)	
AP	0.76 (0.66–0.86)	
S	4.58 (2.96–7.08)	

*Abbreviations*: *T2D* type 2 diabetes, *OR* odds ratio, *CI* confidence interval, *RERI* risk due to interaction, *AP* attributable proportion due to interaction, *S* synergy index

Multivariable analysis adjusted for body mass index (< 18.5, 18.5 ~ 24.0, 24.0 ~ 28.0, and ≥ 28.0 kg/m2), systolic blood pressure (< 140 and ≥ 140 mmHg), low-density lipoprotein cholesterol (< 2.60 and ≥ 2.60 mmol/L), high-density lipoprotein cholesterol (< 1.00 mmol/L in male or < 1.30 mmol/L in female as low level and ≥ 1.00 in male or ≥1.30 in female as high level) and triglyceride (< 1.70 mmol/L and ≥ 1.70 mmol/L); significant elative excess risk due to interaction (RERI) > 0, attributable proportion due to interaction (AP) > 0, or synergy index (S) > 1 indicates a significant additive interaction

### Sensitive analysis

Excluding patients with diabetes complications or under anti-diabetic medications only slightly changed effects of asparagine to aspartate ratio on diabetes. The addictive interaction of asparagine to aspartate ratio and sex on T2D was significant (Table S1).

Females increased T2D risk only in group with ≥50 years of age (OR, 2.35; 95% CI 1.69–3.28); in male gender, OR (95%CI) of age ≥ 50 vs. < 50 years was 2.64 (1.92–3.62). In female gender, OR (95% CI) of age ≥ 50 vs. < 50 years was 6.65 (3.95–11.2) (Table S2).

### Correlations of asparagine to aspartate ratio with available diabetes traits

Asparagine to aspartate ratio was positively correlated with BMI, SBP, triglyceride, HbA1c (correlation coefficients ranged from 0.07 to 0.15, all *P* values < 0.05), and inversely correlated with HDL-C (correlation coefficients − 0.27, *P* < .0001); Correlations of asparagine to aspartate ratio with LDL-C and duration of diabetes were not statistically significant (Table S3).

## Discussion

In present study, we found that high asparagine to aspartate ratio, i.e., ≥ 1.5, was associated with elevated risk of T2D, and its effect size was greatly enhanced by female gender and > 50 years of age, especially by copresence of both. Our findings showed that abnormal asparagine and aspartate homeostasis contributed to an increased risk of T2D and the abnormal asparagine and aspartate homeostasis had a female gender and older age specific effect on the risk of T2D in Chinese adults.

Only a few studies reported inconclusive findings regarding the associations of asparagine and aspartate with the risk of T2D. In subjects without diabetes and cardiovascular diseases, asparagine was inversely correlated with fasting insulin and insulin resistance while aspartate was inversely correlated with plasma glucose [16]. Two prospective observational studies in the USA found that baseline plasma asparagine was a protective marker of diabetes [28, 29]. In these research studies, findings about aspartate were accordant with ours, but results about asparagine were opposite to ours. The discrepancy may derive from stage of sampling: asparagine is important precursor for many other amino acids and thus has abundant functions. Imbalanced nutrition consumption and endoplasmic reticulum stress, which are risk factors for diabetes, can accelerate asparagine consumption and activate asparagine synthetase gene (ASNS) subsequently [30]. Potential explanation for this is that before the onset of diabetes, asparagine is likely depleted whereas ASNS remains the same; then in later period, persistent adverse stimulation may upregulate ASNS and result in asparagine overload and aspartate deficiency. Notably, in our study, patients with longer duration of diabetes did not have higher asparagine to aspartate ratio compared with patients with shorter duration of diabetes. We speculated that upregulate ASNS may accelerate process of hospitalization. So, it is reasonable that patients with different duration of diabetes have the same asparagine to aspartate ratio level. In this regard, information about individual differences in gene expression will be valuable. We cannot exclude asparaginase downregulation although limited published literature investigated impact of environment on asparaginase. Given that asparaginase can be produced by Escherichia coli, our study may provide clues linking gut microbiota and diabetes [12].

Asparagine can also suppress phosphorylation of AMPK and upregulate mTORC1 [15, 31], which can lead to increased insulin resistance and reduced β cell reserve [32]. Our study also indicates that low aspartate could lead to T2D; nevertheless, the mechanistic studies are lacking to explain this association. In the current study, we further found that abnormal asparagine and aspartate homeostasis had a gender and age specific effect on the risk of T2D and female gender and older age (defined as over 50 years of age), particularly, copresence of female and older age, greatly amplified the effect of abnormal asparagine and aspartate homeostasis on the risk of T2D. Abnormal asparagine and aspartate homeostasis explained some of elevated risk in females aged greater than 50 years. One acknowledged assumption of higher risk of T2D for females at later adult life was hormonal transition of menopause, i.e., estrogen depletion [6, 18]. Previous, estrogen was known as an important regulator of metabolic status [33]. Obesity was accelerated in individuals with lower levels of estrogen. Estrogen deficiency can also induce insulin resistance, decreasing insulin releasing and β-cell apoptosis [18]. In this connection, randomized controlled trials showed that menopausal hormone therapy delayed the onset of T2D in women [34]. Our results further supports that hormonal transition at postmenopausal period may play a role in the different effect of asparagine and aspartate homeostasis for the risk of T2D. It is of interest to examine evidence about interactions of asparagine and aspartate homeostasis with estrogen for T2D. Estrogen exposure upregulated AMPK activity in skeletal muscle of ovariectomized mice [35]. When culturing myotubes and adipocytes with and without estrogen, estrogen activated AMPK and suppressed mTORC1 subsequently dependent on nutrient availability [36]. So, it was plausible that at some condition, e.g., nutrition unavailability, estrogen deficiency suppressed AMPK and upregulated mTORC1, which is consistent with the observed effects of asparagine supplement for T2D [14, 15]. Therefore, we speculate that coexistence of abnormal asparagine and aspartate homeostasis and estrogen deficiency accelerate the progress of insulin resistance and promoted onset of T2D via AMPK-mTORC1 pathway. In other words, estrogen deficiency influences metabolism based on asparagine and aspartate homeostasis. Nevertheless, we could not exclude other mechanisms given to the complex function of estrogen and limited knowledge of mechanisms in relation to abnormal asparagine and aspartate homeostasis and metabolic disorders. Some studies found that mechanism of estrogen on glucose homeostasis may independent of insulin action [37], so maybe amino acids metabolism can provide some clues for this.

Our study had important implications for both clinic and basic science. The incidence of T2D differs between males and females, but the reasons for this disparity remain elusive. Our study helps understand why females after menopause had increased risk of metabolic disease including T2D. Postmenopausal females with high asparagine to aspartate ratio had higher risk of T2D, which deserves more attention. Future physiological investigations into this mechanism are warranted to better understand the molecular mechanism and find possible ways to reduce disease burden of T2D in this high risk group. Our study had several limitations. First, our study was a retrospective cross-sectional survey, thus the causal relationship cannot be established and prospective cohorts are needed to confirm the findings of our study. Second, our subjects were in-patients with relatively severe condition and acute unwell T2D. Sensitive analysis (excluding patients with diabetes complications) removed influence of acute events related to macro- and micro vascular complications, whereas we cannot exclude effects of other possible acute events on metabolism. Research in general populations are warranted to confirm our findings. Third, there were more proportion of males than females, so there might be some selective bias in our study. Prospective studies are thus warranted. Fourth, we did not measure estrogen in our patients, so we could not examine associations of amino acids and estrogen. Fifth, although most women experience menopause at around 50 years old, we did not collect the accurate age of menopause. So, we cannot know the distribution of pre- and post-menopaused individual in our cohort. Indeed, whether women that underwent menopause earlier in life have increased asparagine to aspartate ratio is of significance and remains to be determined.

In conclusion, we found that high asparagine to aspartate ratio was associated with elevated risk of T2D. The effect of high asparagine and aspartate ratio was greatly enhanced by female gender and being greater than 50 years of age, especially by copresence of both. Our findings suggest that abnormal asparagine and aspartate homeostasis at postmenopausal period may be one of the reasons for the increased risk of T2D in the high T2D-risk group. Further investigations into the underlying molecular mechanisms are warranted for better understanding of the cause of T2D and possible prevention of T2D in the high risk group.

### Perspectives and significance

Sex difference exists along life span although specific mechanism and consequence have not been figured out. However, plenty of previous studies implied possible relationships between biological sex and metabolic disease including T2D. Our study provided additional clues about why females after menopause had increased risk of metabolic disease including T2D, which was meaningful for personalize management of T2D especially for postmenopausal women. In the future, prospective studies were warranted to replicate our findings and more mechanistic investigations are needed to elucidate the molecular mechanisms for identification of more efficient and personalized therapeutic targets for males and females in different life course.

## Supplementary information

**Additional file 1:** **Table** S1. Interactive effects of asparagine and aspartate homeostasis with sex in patients without diabetes complications or use of anti-diabetic medications. **Table S2.** Effects sex on T2D at different age groups and effects of age on T2D at different sex. **Table S3.** Partial correlations^$^ of asparagine to aspartate ratio with available diabetes traits. **Figure** S1**.** Odds ratio of asparagine or aspartate alone for T2D risk.

## Data Availability

Data are available on request to the corresponding author.
