# Supplementary material for: Interactive effects of asparagine and aspartate homeostasis with sex and age for the risk of type 2 diabetes risk
Source: Biol Sex Differ. 2020 Oct 22;11:58. doi: 10.1186/s13293-020-00328-1 (PMC7579815; doi:10.1186/s13293-020-00328-1)

**Table S1.** Interactive effects of asparagine and aspartate homeostasis with sex in patients without diabetes complications or use of anti-diabetic medications .

|  | Univariable model | |  | Multivariable model | |
| --- | --- | --- | --- | --- | --- |
|  | OR (95%CI) | P-value |  | OR (95%CI) | P-value |
| **Excluding patients with diabetes complications (N=565)** | | | | | |
| Asn: Asp >1.5 vs. ≤ 1.5 | 7.88 (5.14-12.1) | <.0001 |  | 8.26 (4.99-13.6) | <.0001 |
| Asn: Asp ≤1.5 & male | Reference |  |  | Reference |  |
| Asn: Asp ≤1.5 & female | 1.95 (0.86-4.43) | 0.1091 |  | 1.21 (0.48-3.04) | 0.6939 |
| Asn: Asp >1.5 & male | 7.25 (3.98-13.2) | <.0001 |  | 6.83 (3.51-13.3) | <.0001 |
| Asn: Asp >1.5 & female | 22.3 (12.1-41.2) | <.0001 |  | 12.3 (6.07-24.9) | <.0001 |
| Interaction measure |  |  |  |  |  |
| RERI | 14.1 (4.99-23.2) |  |  | 5.26 (0.23-10.3) |  |
| AP | 0.63 (0.52-0.74) |  |  | 0.42 (0.19-0.66) |  |
| S | 2.95 (2.08-4.19) |  |  | 1.87 (1.17-2.99) |  |
| **Excluding patients under anti-diabetic medications (N=553)** | | | | | |
| Asn: Asp >1.5 vs. ≤ 1.5 | 5.25 (3.61-7.64) | <.0001 |  | 6.33 (3.95-10.2) | <.0001 |
| Asn: Asp ≤1.5 & male | Reference |  |  | Reference |  |
| Asn: Asp ≤1.5 & female | 1.63 (0.80-3.30) | 0.1765 |  | 1.37 (0.58-3.23) | 0.4759 |
| Asn: Asp >1.5 & male | 4.43 (2.68-7.34) | <.0001 |  | 5.39 (2.93-9.91) | <.0001 |
| Asn: Asp >1.5 & female | 13.6 (8.04-22.9) | <.0001 |  | 10.7 (5.58-20.6) | <.0001 |
| Interaction measure |  |  |  |  |  |
| RERI | 8.51 (3.71-13.3) |  |  | 4.95 (0.54-9.36) |  |
| AP | 0.63 (0.50-0.75) |  |  | 0.46 (0.23-0.69) |  |
| S | 3.09 (2.03-4.71) |  |  | 2.04 (1.23-3.39) |  |

Abbreviations: T2D, type 2 diabetes; OR, odds ratio; CI, confidence interval, Asn, asparagine; Asp, aspartate; RERI, risk due to interaction; AP, attributable proportion due to interaction; S, synergy index;

Multivariable model adjusted for body mass index (<18.5, 18.5~24.0, 24.0~28.0 and ≥28.0 kg/m2), systolic blood pressure (<140 and ≥140 mmHg), low-density lipoprotein cholesterol (<2.60 and ≥2.60 mmol/L) , high-density lipoprotein cholesterol (<1.00 mmol/L in male or <1.30 mmol/L in female as low level and ≥1.00 in male or ≥1.30 in female as high level) and triglyceride (<1.70 mmol/L and ≥1.70 mmol/L); Significant elative excess risk due to interaction (RERI) >0, attributable proportion due to interaction (AP) >0 or synergy index (S) >1 indicates a significant additive interaction.

**Table S2**. Effects sex on T2D at different age groups and effects of age on T2D at different sex.

|  | Univariable model | |  | Multivariable model | |
| --- | --- | --- | --- | --- | --- |
|  | OR (95%CI) | P-value |  | OR (95%CI) | P-value |
| **Effects sex on T2D at different age groups** | | | | | |
| Age < 50 years |  |  |  |  |  |
| Males (72.2%) | Reference | 0.3617 |  | Reference | 0.5544 |
| Females (27.8%) | 1.18 (0.83-1.69) |  |  | 0.86 (0.52-1.42) |  |
| Age ≥ 50 years |  |  |  |  |  |
| Males (60.7%) | Reference | <.0001 |  | Reference | <.0001 |
| Females (39.3%) | 3.45 (2.70-4.41) |  |  | 2.35 (1.69-3.28) |  |
| **Effects of age on T2D at different sex** | | | | | |
| Males |  |  |  |  |  |
| Age < 50 years (48.5%) | Reference | <.0001 |  | Reference | <.0001 |
| Age ≥ 50 years (51.5%) | 3.06 (2.39-3.91) |  |  | 2.64 (1.92-3.62) |  |
| Females |  |  |  |  |  |
| Age < 50 years (35.9%) | Reference | <.0001 |  | Reference | <.0001 |
| Age ≥ 50 years (64.1%) | 8.94 (6.26-12.8) |  |  | 6.65 (3.95-11.2) |  |

Abbreviations: T2D, type 2 diabetes; OR, odds ratio; CI, confidence interval;

Multivariable model adjusted for body mass index (<18.5, 18.5~24.0, 24.0~28.0 and ≥28.0 kg/m2), systolic blood pressure (<140 and ≥140 mmHg), low-density lipoprotein cholesterol (<2.60 and ≥2.60 mmol/L) , high-density lipoprotein cholesterol (<1.00 mmol/L in male or <1.30 mmol/L in female as low level and ≥1.00 in male or ≥1.30 in female as high level) and triglyceride (<1.70 mmol/L and ≥1.70 mmol/L).

Table S3. Partial correlations^$^ of asparagine to aspartate ratio with available diabetes traits.

|  | N | correlation coefficient | P-value |
| --- | --- | --- | --- |
| Body mass index * | 2554 | 0.14 | <.0001 |
| Systolic blood pressure * | 2554 | 0.07 | 0.0005 |
| Triglyceride ^#^ | 2250 | 0.15 | <.0001 |
| LDL-C * | 2250 | -0.04 | 0.0533 |
| HDL-C * | 2250 | -0.27 | <.0001 |
| HbA1c ^#^ | 631 | 0.09 | 0.0437 |
| Duration of diabetes ^#^ | 1027 | -0.02 | 0.6695 |

Abbreviations: LDL-C, low-density lipoprotein cholesterol; HDL-C, high-density lipoprotein cholesterol; HbA1c, glycated hemoglobin;

$, adjusted for age and sex;

*, correlation coefficients were derived from Pearson correlation;

#, correlation coefficients were derived from Spearman correlation.

**Figure S1**. Odds ratio of asparagine or aspartate alone for T2D risk.

Abbreviations: T2D, type 2 diabetes; Asn, asparagine; Asp, aspartate;

A, Odds ratio of asparagine for T2D risk; B, Odds ratio aspartate for T2D risk.

The black curve was derived from univariable analysis, and the blue curve derived from multivariate analysis that adjusted for age, sex, body mass index (<18.5, 18.5~24.0, 24.0~28.0 and ≥28.0 kg/m2), systolic blood pressure (<140 and ≥140 mmHg), low-density lipoprotein cholesterol (<2.60 and ≥2.60 mmol/L) , high-density lipoprotein cholesterol (<1.00 mmol/L in male or <1.30 mmol/L in female as low level and ≥1.00 in male or ≥1.30 in female as high level) and triglyceride (<1.70 mmol/L and ≥1.70 mmol/L); The red curve stands for the reference level (i.e., the odds ratio for type 2 diabetes mellitus was 1)


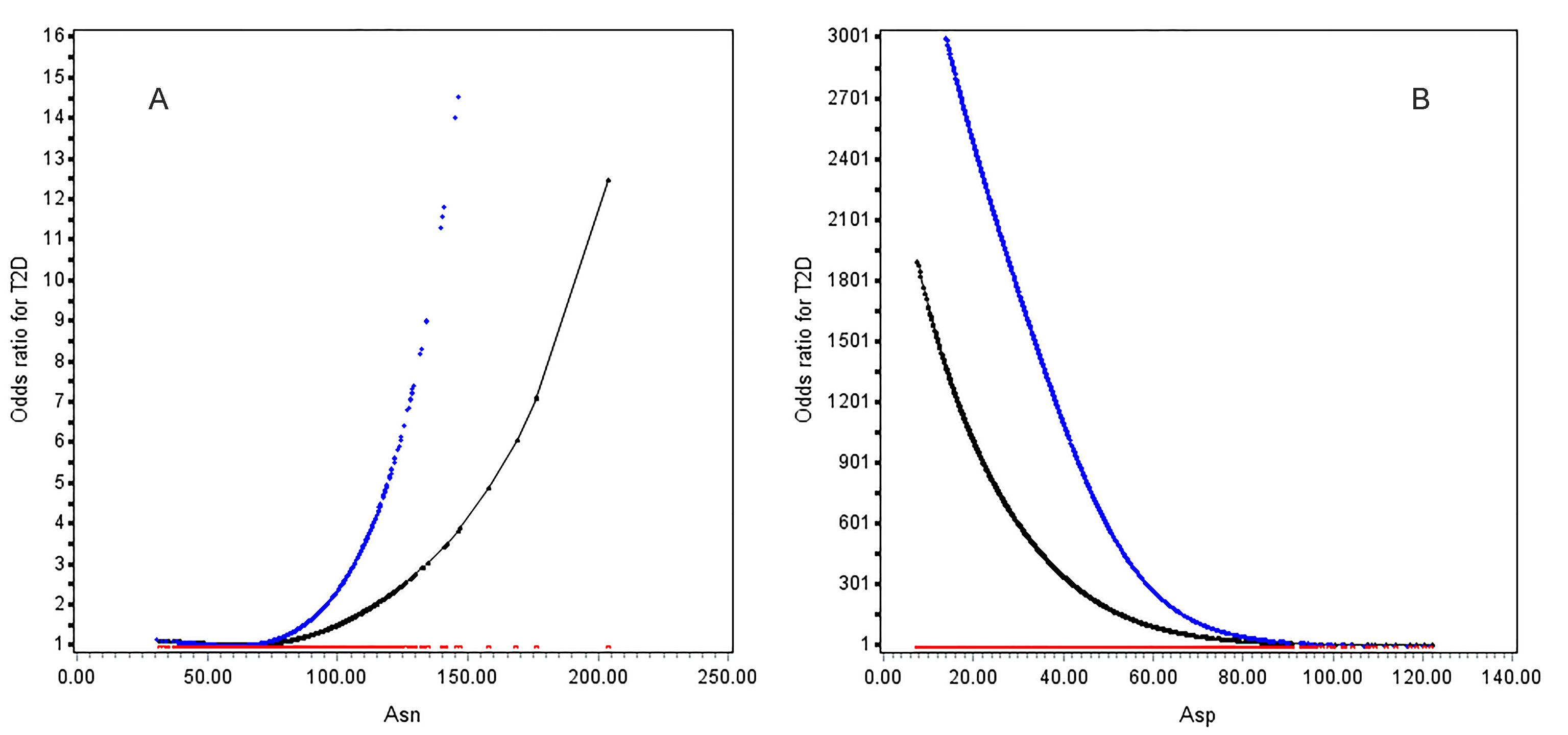

Supplement: Supplementary file 1 — Additional file 1: Table S1. Interactive effects of asparagine and aspartate homeostasis with sex in patients without diabetes complications or use of anti-diabetic medications. Table S2. Effects sex on T2D at different age groups and effects of age on T2D at different sex. Table S3. Partial correlations$ of asparagine to aspartate ratio with available diabetes traits. Figure S1. Odds ratio of asparagine or aspartate alone for T2D risk. [file 13293_2020_328_MOESM1_ESM.doc]
